# Epidemiología de una Unidad de Cuidado Intensivo Cardiovascular de Referencia Nacional. Resultados del Registro RECICA-INCOR

**DOI:** 10.47487/apcyccv.v1i1.9

**Published:** 2020-03-30

**Authors:** David Miranda, Ofelia Aráoz, Maritza Rosales, Rosario Guzmán

**Affiliations:** 1 Servicio de Cardiología Clínica - Instituto Nacional Cardiovascular - INCOR EsSalud. Lima, Perú. Servicio de Cardiología Clínica Instituto Nacional Cardiovascular - INCOR EsSalud Lima Perú

**Keywords:** cuidado intensivo cardiovascular, epidemiología, Perú, cardiovascular intensive care, epidemiology, Peru

## Abstract

**Antecedentes:**

Las unidades de cuidado intensivo cardiovascular han evolucionado en el tiempo, pasando de ser unidades dedicadas exclusivamente a pacientes post infarto de miocardio a albergar patologías cardiovasculares complejas y variadas. No tenemos en el Perú datos sobre las características de los pacientes en estas unidades.

**Método:**

Evaluación prospectiva de las características clínico epidemiológicas de pacientes que ingresaron entre julio y noviembre de 2018 a la unidad de cuidado intensivo e intermedio del Instituto Nacional Cardiovascular INCOR en Lima, Perú.

**Resultados:**

Se incluyó a 199 pacientes. La mediana de edad del grupo fue de 67 años, 20% mayores a 80 años y 75.8 % de sexo masculino. 60% de los casos provenían de la unidad de emergencia. Los diagnósticos de ingreso más frecuentes fueron los síndromes coronarios agudos (SCA) (35%) y la falla cardíaca agudamente descompensada (20%). La mortalidad intrahospitalaria fue de 4.5%, siendo mayor (12%) en los pacientes con reingreso a cuidado intensivo.

**Conclusión:**

En este primer registro de cuidado cardiaco crítico en el Perú, el SCA fue la principal causa de ingreso seguido de la falla cardíaca agudamente descompensada. La mortalidad intrahospitalaria fue mayor en pacientes con reingreso a cuidado intensivo.

## Abstract (en)

**Background:**

Cardiovascular intensive care units have evolved in time, from being exclusively dedicated to the care of myocardial infarction patients, to treating complex and varied cardiovascular pathologies. We do not have data about the characteristics of patients in cardiovascular intensive care units in Peru.

**Material and Methods:**

We prospectively evaluated the clinical and epidemiological characteristics of patients admitted between July and November 2018 to the intensive and intermediate care unit of the National Cardiovascular Institute INCOR in Lima, Peru.

**Results:**

A total of 199 patients were enrolled in the study. The median age was 67 years, 20% older than 80 years and 75.8% males. 60% of cases they came from the emergency unit. The most frequent admissions diagnoses were acute coronary syndromes (ACS) (35%) and acutely decompensated heart failure (20%). In-hospital mortality was 4.5%, higher (12%) in patients with readmissions to intensive care.

**Conclusions:**

In this first registry of cardiac critical care in Peru, ACS continues to be the main cause of admission followed by acutely decompensated heart failure. The in-hospital mortality was higher in patients with readmissions to the intensive care unit.

Como respuesta al incremento de la enfermedad cardiovascular aguda representada por el infarto de miocardio, surgieron a inicios de la década de 1960 las unidades de cuidado coronario (UCC) con el trabajo del Dr. Desmond Julian.^1^^)^ La atención de los pacientes en las primeras UCC consiguió la reducción de la mortalidad de 30 a 16% y el desarrollo posterior de nuevas terapias para el infarto redujo aún más la mortalidad hasta 7%. ^2^^-^^4^

Instalado y demostrado el beneficio del cuidado coronario agudo hemos sido testigos del cambio epidemiológico de las UCC. El rol de cuidado post trombólisis coronaria o angioplastia coronaria percutánea se ha modificado y actualmente las unidades especializadas reciben pacientes con síndrome coronario agudo complicado, falla cardíaca aguda, choque cardiogénico, enfermedad cardíaca valvular severa, endocarditis, arritmias ventriculares, entre otros. ^(^^5^^,^^6^^)^ Adicionalmente, ha incrementado la complejidad de los pacientes admitidos pues se han asociado comorbilidades significativas que incrementan la mortalidad y estancia en unidades especializadas. ^(^^7^^,^^8^

Este cambio epidemiológico ha llevado a la reciente publicación de lineamientos para el funcionamiento de las Unidades de Cuidado Intensivo Cardiovascular (UCIC) por parte de las principales sociedades científicas mundiales. ^(^^9^^-^^12^^)^ Estos documentos establecen nuevas definiciones, clasificación de la complejidad de una UCIC, requerimientos para el servicio asistencial, entrenamiento de médicos a cargo y propuestas de investigación en el área.

Ante esta nueva situación, y la ausencia de información local y nacional, consideramos necesario conocer las características y resultados de los pacientes admitidos a la UCIC y a la Unidad de Cuidados Intermedios (UCIN) del Instituto Nacional Cardiovascular INCOR.

## Material y Método

Desarrollamos un estudio prospectivo, descriptivo, unicéntrico, en las unidades de cuidado intensivo y cuidado intermedio cardiovascular de adultos del Instituto Nacional Cardiovascular de EsSALUD en la ciudad de Lima, Perú. Dicho centro hospitalario es el único centro cardiovascular de referencia nacional del sistema de Seguridad Social del Perú (EsSALUD) que solo acepta pacientes referidos de otros centros hospitalarios. Se incluyeron en el estudio todos los pacientes ingresados en las unidades descritas, desde el 1 de julio al 31 de noviembre de 2018; se excluyeron los pacientes que no deseaban participar en el estudio y aquellos pacientes que ingresaron a la unidad de cuidados intensivos postoperatorios de cirugía cardiaca desde su postoperatorio inmediato (aunque si se incluyeron pacientes después del segundo día operatorio que fueron transferidos a UCI clínica por necesidad de espacio en la UCI postoperatoria). Los datos fueron colectados prospectivamente en una base de datos específicamente creada para el estudio mientras el paciente se encontraba en cualquiera de las unidades de cuidado crítico, no se consideró seguimiento luego del alta de estas unidades (salvo reingreso).

Las variables categóricas fueron expresadas en porcentajes, las variables continuas en media y desviación estándar o medianas y rango intercuartil (RIQ) si su distribución no fue normal según el test de normalidad de Shapiro-Wilk. El análisis estadístico se realizó con el programa STATA 14.0.

## Resultados

De julio a noviembre de 2018, ciento noventa y nueve pacientes ingresaron a las unidades de cuidado crítico clínico de adultos de INCOR: 105 a UCIC y 94 a UCIN, quienes representan la población de estudio.

### Población

**Figura 1:**
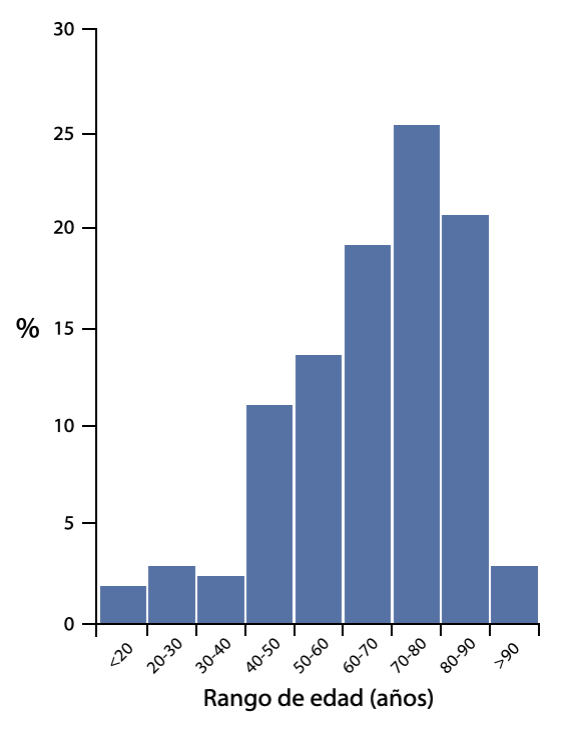
Distribución según edad.

En su mayoría los pacientes fueron del sexo masculino, con una mediana de edad de 67 años, rango intercuartil (RIQ) de 55 a 79, y 25% con edad de 80 años a más. (Figura 1) La mediana de edad en UCIC fue de 66 años (RIQ: 56 -79) y en UCIN de 70 años (RIQ: 54-79). El 81% de ingresos presentaron alguna comorbilidad asociada. (Tabla 1) Un 59.4% fueron ingresados provenientes de sala de emergencia o cuidado cardiaco agudo (UCCA), 18.5% de sala de cardiología invasiva (laboratorio de cateterismo cardiaco y de electrofisiología), 8% de hospitalización general, 7.5% de la unidad de cuidado intensivo post operatorio de cirugía cardiaca (UCI PO) y el 6.5% restante ingreso a UCIC por descompensación proveniente de UCIN.

**Tabla 1:** Características de la población de estudio según localización

		UCIC (n=105)		UCIN (n=94)		Total (N=199)	
Sexo	Masculino	76	(72.3)	75	(79.7)	151	(75.8)
Procedencia	UCCA	54	(51.4)	64	(68.1)	118	(59.4)
	Hemodinámica	22	(21.0)	15	(16.0)	37	(18.5)
	UCI PO	13	(12.4)	2	(2.1)	15	(7.5)
	Hospitalización	3	(2.8)	13	(13.8)	16	(8.0)
	UCIN	13	(12.4)	-	-	13	(6.5)
Rango edad	< 45 años	11	(10.7)	14	(15.4)	25	(12.9)
	46- 60 años	24	(23.3)	18	(19.8)	42	(21.6)
	61 a 80 años	48	(46.6)	42	(46.1)	90	(46.4)
	> 80 años	22	(20.9)	20	(18.7)	42	(21.1)
Antecedente	HTA	69	(65.7)	54	(57.4)	123	(61.8)
	DM	27	(25.7)	23	(24.4)	50	(25.1)
	Tabaco	24	(22.8)	24	(25.5)	48	(24.1)
	Dislipidemia	24	(22.8)	24	(25.5)	48	(24.1)
	EPOC	5	(4.7)	2	(2.1)	7	(3.5)
	IRC III/IV	3	(2.8)	11	(11.7)	14	(7.0)
	Cáncer	1	(0.9)	1	(1.1)	2	(1.0)
	Infarto previo	24	(22.8)	23	(24.4)	47	(23.6)
	Insuficiencia cardíaca	19	(18.1)	15	(15.9)	34	(17.0)
	Stroke	5	(4.8)	9	(9.6)	14	(7.0)
	Revascularizado	9	(8.6)	16	(17.0)	25	(12.5)
	Fibrilación atrial	11	(10.5)	17	(18.1)	28	(14.0)
	DAI/TRC	1	(0.9)	0	(0)	1	(0.5)
	Trasplante cardíaco	0	(0)	1	(1.1)	1	(0.5)
	Prótesis valvular	6	(5.7)	4	(4.2)	10	(5.0)

Se reporta frecuencias (y porcentajes) para cada variable.

UCCA: unidad de cuidado cardiaco agudo; UCI PO: unidad de cuidado crítico post cirugía cardiaca;

UCIN: unidad de cuidado intermedio; UCIC: unidad de cuidado intensivo cardiológico; HTA: hipertensión arterial;

DM: diabetes mellitus; EPOC: enfermedad pulmonar obstructiva crónica; IRC: insuficiencia renal crónica;

DAI/TRC: cardiodesfibrilador implantable/terapia de resincronización cardíaca

### Diagnósticos, procedimientos y tratamientos

**Figura 2:**
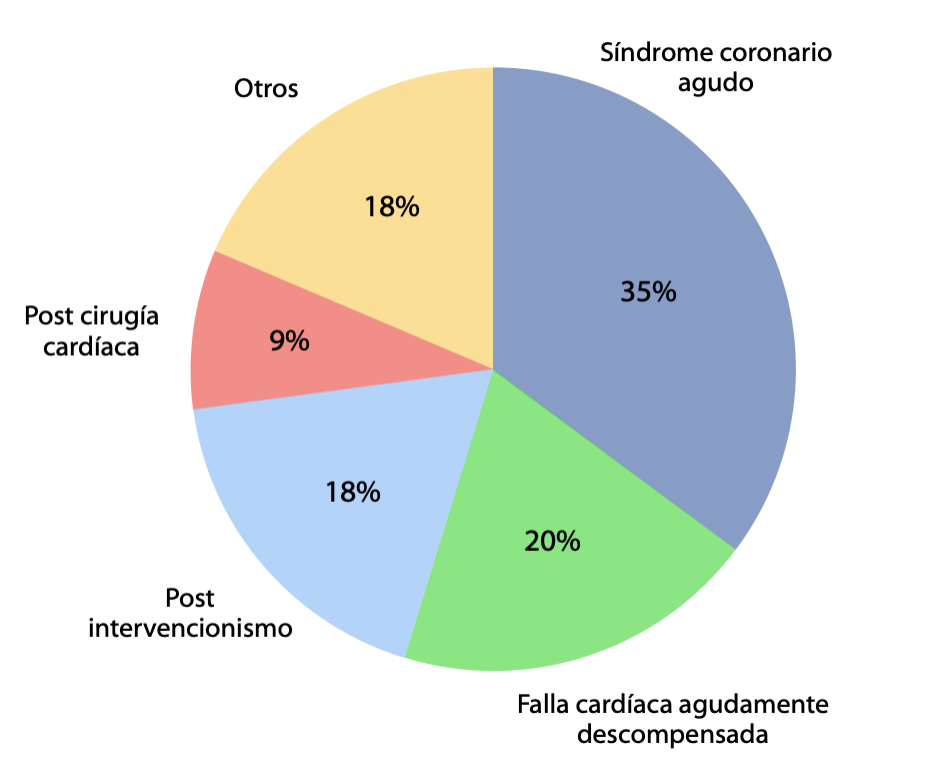
Motivos de ingreso a cuidados críticos cardiológicos.

El motivo principal de ingreso fue el síndrome coronario agudo (SCA) (35.2%) en sus diferentes presentaciones, seguido del diagnóstico de falla cardiaca agudamente descompensada (19.6%). (Figura 2) Los diagnósticos según la unidad de ingreso se presentan en la Tabla 2. 

**Tabla 2:** Diagnósticos de Ingreso

	UCIC (n = 105)		UCIN (n = 94)		TOTAL (n = 199)	
Angina Inestable	2	(1.9)	2	(2.1)	4	(2.0)
Arritmia cardiaca	1	(0.9)	6	(6.4)	7	(3.5)
Choque cardiogénico	7	(6.7)	0	(0)	7	(3.5)
Complicación post intervencionismo	4	(3.8)	0	(0)	4	(2.01)
Endocarditis Infecciosa	2	(1.9)	2	(2.1)	4	(2.01)
Falla cardiaca avanzada	4	(3.8)	1	(1.1)	5	(2.5)
Falla cardiaca crónica descompensada	17	(16.2)	22	(23.4)	39	(19.6)
Infarto de miocardio ST elevado	23	(21.9)	24	(25.5)	47	(23.6)
Infarto de miocardio ST no elevado	5	(4.8)	14	(14.9)	19	(9.6)
Insuficiencia respiratoria	2	(1.9)	1	(1.1)	3	(1.5)
Post intervencionismo no complicado	18	(17.1)	14	(14.9)	32	(16.1)
Post operado de cirugía cardiaca	13	(12.4)	4	(4.2)	17	(8.5)
Recuperado de arresto cardiaco	4	(3.8)	0	(0)	4	(2.0)
Síncope	0	(0)	3	(3.2)	3	(1.5)
Síndrome aórtico agudo	3	(2.9)	1	(1.1)	4	(2.0)

Se reporta frecuencias (y porcentajes) para cada variable.

En caso de SCA la mediana de edad fue de 72 años (RIQ: 62-79), de la estancia hospitalaria en cuidados intensivos fue 5 días (RIQ:3-7) y la mortalidad intrahospitalaria fue de 6.6%. En el caso de falla cardiaca agudamente descompensada la mediana de edad fue de 62 años (RIQ: 44-75), de la estancia hospitalaria fue 7 días (RIQ: 3-10) y la mortalidad intrahospitalaria 7.7%.

Diferentes procedimientos médicos se realizaron en los pacientes, entre los que se incluyen: ecocardiografías (89%, divididas en 81% transtorácicas y 8% transesofágicas), ingreso a sala de cateterismo cardiaco para coronariografía con o sin angioplastia (28%), colocación de catéter venoso central (31.7%), colocación de sonda vesical (24%), colocación de línea arterial (20%), ventilación mecánica invasiva y no invasiva (19%), colocación y(o) monitoreo de marcapaso temporal (12%), intubación orotraqueal (9%), reanimación cardiopulmonar avanzada (6.6%), monitoreo invasivo de presión de arteria pulmonar (5%, de este grupo el 60% por choque cardiogénico), colocación de balón de contrapulsación intra-aórtico (5%), toracocentesis (3.5%) y cardioversión eléctrica (2.5%). 

Las medicinas administradas con más frecuencia fueron los antiagregantes plaquetarios (68.5%) y diuréticos (58.7%) (Tabla 3). El uso de inotrópicos en UCIC llego a 36% y de nitratos a 45%; la dobutamina fue el inotrópico más utilizado (72%) con una mediana de tiempo de uso de 3 días (RIQ 2 -5) y un rango de 1 a 49 días. 

**Tabla 3:** Medicación administrada

	UCIC (n = 105)		UCIN (n = 94)		Total (n = 199)	
Diuréticos	67	(63.8)	50	(53.2)	117	(58.7)
Inotrópicos	38	(36.2)	11	(11.7)	49	(24.6)
Nitratos	47	(44.8)	27	(29.0)	74	(37.3)
Vasopresores	28	(27.0)	4	(4.3)	32	(16.2)
Insulina	12	(11.6)	14	(15.2)	26	(13.3)
Antiagregantes	74	(71.2)	61	(65.6)	135	(68.5)
Estatinas	46	(44.2)	58	(62.3)	104	(52.7)
IECA /ARA2	32	(31.7)	42	(46.1)	74	(38.5)
Betabloqueadores	25	(25.5)	43	(47.2)	68	(35.9)
Anticoagulantes	50	(48.5)	29	(30.9)	79	(40.1)
Hemoderivados	7	(6.8)	3	(3.3)	10	(5.1)

Se reporta frecuencias (y porcentajes) para cada variable.

IECA/ARA2: Inhibidores de la enzima convertidora de angiotensina/ antagonistas del receptor de angiotensina 2.

Treinta y siete pacientes (19% de la población) estuvieron con ventilación mecánica (invasiva 83% y no invasiva 17%), 92% de ellos en UCIC y 8% en UCIN; en estos pacientes, la mediana de días en ventilación mecánica fue de 2 días (RIQ: 1- 5) con un rango de 1 a 39 días. El motivo principal de ingreso a ventilación mecánica fue post reanimación cardiopulmonar 27%, por insuficiencia respiratoria 27%, post cirugía/intervencionismo 19%, choque cardiogénico 11%, falla cardíaca 8% y otros 8%. El modo de inicio de la ventilación mecánica preferido fue asistido/controlado por presión (47%). 

La mediana de estancia hospitalaria fue de 5 días (RIQ: 2 - 8), con un rango de 1 a 87 días, pero en pacientes con ventilación mecánica esta fue de 9 días (RIQ: 5-16) a diferencia de los pacientes sin ventilación que fue de 4 días (RIQ: 2-7). La estancia hospitalaria según diagnóstico se presenta en la Tabla 4, donde la insuficiencia respiratoria (3 casos) tuvo la mayor estancia por procesos de infección y ventilación mecánica prolongada. El destino del paciente al alta de las unidades críticas fue como sigue: hospitalización 49%, su casa 29%, cirugía 13%, morgue 4.5%, otro hospital 2%, UCCA 1.5% y UCI PO 0.5%.

**Tabla 4:** Estancia hospitalaria (en días) en unidades críticas según diagnóstico de ingreso

Insuficiencia respiratoria	68	(12 - 77)
Recuperado de arresto cardiaco	12.5	(6.5 - 62.5)
Falla cardiaca avanzada	10	(10 - 16)
Falla cardiaca agudamente descompensada	7	(3 -10)
Endocarditis infecciosa	5.5	(3 - 6.5)
Choque cardiogénico	5	(4 - 14)
Infarto de miocardio ST elevado	5	(2 - 7)
Post operado de cirugía cardiaca	5	(4 - 17)
Síncope	5	(1 - 9)
Infarto de miocardio ST no elevado	4.5	(3 - 6)
Complicación post intervencionismo	3.5	(2.5 - 5)
Angina Inestable	3	(2 - 4.5)
Arritmia cardiaca	2	(1 - 5)
Post intervencionismo	2	(1 - 4.5)
Síndrome aórtico agudo	1	(1 - 3.5)

Se reporta mediana (y rango intercuartil) para cada variable.

### Complicaciones y mortalidad intrahospitalaria

La mortalidad intrahospitalaria fue de 4.5% (9 pacientes), 5.7% en UCIC y 3.2% en UCIN, los diagnósticos de ingreso a UCIC/UCIN con mayor porcentaje de mortalidad intrahospitalaria se presentan en la Tabla 5. 

**Tabla 5:** Mortalidad intrahospitalaria según diagnóstico de ingreso a la unidad

Shock cardiogénico	28.6%
Falla cardíaca avanzada	20.0%
Falla cardiaca agudamente descompensada	7.7%
Síndrome coronario agudo	7.3%
Recuperado de arresto cardíaco	25.0%

Entre las complicaciones más frecuentes encontramos: infecciones (8.6%), insuficiencia renal aguda AKIN 3 (4.5%), sangrado mayor (intracerebral, intraperitoneal, disminución de hemoglobina < 12 mg/dl) (3%) y evento vascular cerebral agudo (2.5%). En cuanto a las infecciones intrahospitalarias: 35% fueron de origen pulmonar, 24% urinario, 12% dérmico, 12% hematógeno, 11% vías aéreas altas y 6% desconocido.

Un subgrupo especialmente susceptible fueron 16 pacientes (8% del total) que ingresaron a UCIC provenientes de UCIN u hospitalización. Los diagnósticos fueron falla cardiaca descompensada (38%), falla cardiaca avanzada descompensada (19%), post reanimación cardiopulmonar (19%), complicación post intervencionismo (12%) e insuficiencia respiratoria (12%). En este grupo la mortalidad intrahospitalaria fue 12.5% y las infecciones 31%.

En cuanto a las principales complicaciones por patología encontramos que en los pacientes con SCA el 6.5% se complicó con infecciones, 5.1% con falla renal (AKIN II-III) y 1.3 % con evento vascular cerebral agudo. En los pacientes hospitalizados por falla cardiaca descompensada el 11.7% se complicó con infecciones, 9.8% con falla renal (AKIN II-III) y 1.9% con evento vascular cerebral agudo, además en estos pacientes la presencia de infección aumento la estancia hospitalaria de un promedio de 7 días en los pacientes sin infección a 24 días en los infectados.

En este primer registro de pacientes en una unidad de cuidado crítico cardiovascular en el Perú, encontramos que los síndrome coronarios agudos siguen ocupando un lugar primordial como causa de ingreso.

## Discusión

La población estudiada comprendió principalmente adultos mayores de sexo masculino como ocurre en los más importantes registros internacionales de UCIC de alta complejidad. ^(^^13^^-^^16^^)^ A pesar del incremento de la edad de la población atendida en nuestro hospital, la proporción de mayores de 80 años fue menor a la mostrada en los registros francés de Roubille et al. ^(^^17^ e italiano de Casella et al. ^(^^5^ (42.1% y 39% respectivamente) en concordancia con el mayor promedio de edad de la población europea respecto a la población latinoamericana.

El factor de riesgo cardiovascular más prevalente en los pacientes fue la hipertensión arterial seguido de la diabetes mellitus tipo 2, patrón semejante a reportes internacionales. ^(^^5^^,^^13^^,^^16^^)^ La presencia de comorbilidades como enfermedades pulmonares obstructivas crónicas (EPOC) e insuficiencia renal crónica se encuentra por debajo de los niveles reportados en registros europeos^5^^,^^15^^)^ mientras que los antecedentes cardiovasculares más frecuentes fueron enfermedad coronaria y falla cardíaca crónica similar a lo reportado por Gagliardi et al. ^(^^18^

Aunque se ha registrado un cambio epidemiológico en los pacientes admitidos en las UCIC a nivel mundial, el síndrome coronario agudo, principalmente con elevación del segmento ST, sigue siendo la primera causa de admisión a las unidades críticas cardíacas lo que se refleja en nuestra población (28.6%) semejante a lo ocurrido en los registros italiano, alemán, argentino y norteamericano (63.8%, 43.6%, 24.9% y 31.8% respectivamente) ^(^^14^^-^^16^^,^^18^. 

Es importante destacar el aumento del ingreso de pacientes con falla cardíaca agudamente descompensada y para el cuidado de procedimientos complejos de intervencionismo (implante valvular aórtico percutáneo, colocación de endoprótesis aórtica y angioplastia de alto riesgo) de forma similar a lo observado en un reciente registro^16^.

La mortalidad en la UCC reportada en diversos registros varía entre 3.3 y 27.8%^5^^,^^14^^-^^16^^,^^19^ rango en el cual se encuentra la mortalidad de nuestro registro (5.7%). Es mayor en comparación a lo reportado por los registros italianos de Casella et al. y Valente et al. en Italia (3.3 y 4.5% respectivamente) pero menor a la del reciente registro norteamericano de Bohula et al. (8.3%) y el alemán de Zobel et al. (27.8%). Estos hallazgos pueden explicarse por el nivel de complejidad de las UCIC (mortalidad reducida en los registros de UCIC de menor complejidad).

La mortalidad de nuestros pacientes admitidos por SCA fue 6.6% la cual es superior a la reportada en la mayoría de registros salvo el reporte alemán de Zobel et al. (35.9%). Esta diferencia puede ser explicada por varios factores como la forma de admisión de pacientes a INCOR que al ser centro de referencia recibe los síndromes coronarios agudos de alto riesgo, la presencia de UCIN que genera el ingreso a UCIC sólo de los casos más complejos y la mayor severidad de nuestros pacientes. 

La mortalidad por falla cardíaca agudamente descompensada, la segunda causa de admisión a UCIC INCOR, fue similar a la observada en registros mencionados. ^(^^5^^,^^14^^,^^19^ El grupo de mayor riesgo fue aquel que ingresó a UCIC proveniente de UCIN y hospitalización, los que presentaron una alta tasa de mortalidad (12.5%) la cual duplica la mortalidad de UCIC; este hallazgo no ha sido reportado por otros registros.

A diferencia de reportes que muestran aumento de la mortalidad asociada a patología no cardíaca^7^^)^ en unidades críticas, en nuestro registro la patología cardiovascular fue la causa básica de muerte en todos los casos. 

En relación a los procedimientos diagnósticos y terapéuticos realizados en los pacientes, cabe destacar la menor frecuencia de empleo de ventilación mecánica, monitoreo invasivo de presión de arteria pulmonar y soporte circulatorio mecánico en relación a lo reportado por Watson et al. ^(^^13^^)^ y Bohula et al. ^(^^16^^)^ sin embargo la frecuencia de uso es superior a la reportada en registros previos. ^(^^5^^,^^14^^,^^18^^)^ La mayor complejidad de los pacientes admitidos actualmente justifica el incremento de uso de los procedimientos mencionados en nuestro registro. La mayor frecuencia de administración de antiagregantes plaquetarios y diuréticos se justifica por la incidencia de síndrome coronario agudo y falla cardíaca agudamente descompensada en UCIC semejante a lo reportado por Watson et al. ^(^^13^

Las complicaciones más frecuentes fueron las infecciones y la insuficiencia renal aguda lo que confirma la tendencia reportada por Katz et al. en un seguimiento de 17 años de la UCC del hospital universitario de Duke. ^(^^19^^)^ Se observó que la presencia de complicaciones infecciosas y renales son más frecuentes en pacientes ingresados por descompensación de falla cardiaca (estadio C y D) en comparación con otras patologías, lo que nos orienta a mejorar el cuidado de estos pacientes en lo relacionado a la prevención de infecciones respiratorias y dérmicas que aumentan la estancia hospitalaria y los costos asociados a ésta.

### Limitaciones

El presente registro tiene la limitación de ser información reportada por un solo centro de alta complejidad, por lo que no puede ser generalizada a otras unidades del país. Es necesario contar con información a nivel nacional para una mejor caracterización de la atención de nuestros pacientes cardíacos críticos.

## Conclusiones

Este es el primer registro nacional de cuidado crítico de alta complejidad que muestra las características, procedimientos y desenlaces de los pacientes ingresados a la UCIC/UCIN del Instituto Nacional Cardiovascular INCOR, en el cual se evidencian datos similares a unidades críticas de otros países, siendo los síndromes coronarios agudos la principal etiología de ingreso, seguido de la insuficiencia cardíaca agudamente descompensada. 

La mortalidad intrahospitalaria fue mayor en pacientes con reingresos a UCIC por complicaciones luego de haber sido transferidos a unidades de menor cuidado y en pacientes con diagnóstico de choque cardiogénico.

## Financiación:

Ninguna
